# Health care provider trust in vaccination: a systematic review and qualitative meta-synthesis

**DOI:** 10.1093/eurpub/ckab209

**Published:** 2022-01-11

**Authors:** Mobeen Ahmad, Adebisi Akande, Umair Majid

**Affiliations:** 1 Department of Internal Medicine, Abington Memorial Hospital/Abington-Jefferson Health, Abington, PA, USA; 2 Department of Biological Sciences, University of Toronto, Toronto, ON, Canada; 3 Institute of Health Policy, Management and Evaluation, University of Toronto, Toronto, ON, Canada

## Abstract

**Background:**

Vaccine hesitancy is a growing issue globally amongst various populations, including health care providers. This study explores the factors that influence vaccine hesitancy amongst nurses and physicians.

**Methods:**

We performed a qualitative meta-synthesis of 22 qualitative and mixed-method studies exploring the factors that may contribute to vaccine hesitancy amongst nurses and physicians. We included all articles that mentioned any aspect of trust concerning vaccination, including how trust may influence or contribute to vaccine hesitancy in nurses and physicians.

**Results:**

Our findings revealed that vaccine hesitancy amongst nurses stemmed predominantly from two factors: distrust in health authorities and their employers, and distrust in vaccine efficacy and safety. Both nurses and physicians had a precarious relationship with health authorities. Nurses felt that their employers and health authorities did not prioritize their health over patients’ health, provided inaccurate and inconsistent vaccine information, and were mistrustful of pharmaceutical company motives. Like nurses, physicians were also skeptical of pharmaceutical company motives when it came to vaccination. Additionally, physicians also held doubts regarding vaccine efficacy and safety.

**Conclusions:**

The relationship health care providers or their patients have with health authorities and other providers regarding vaccination serves as unsystematic clinical experiences that may bolster vaccine hesitancy. Providing accurate and tangible information to emphasize the safety and efficacy of vaccines to health care providers may help address their specific concerns that may ultimately increase vaccine uptake.

## Introduction

Vaccination is among the most significant public health interventions in modern medicine. Vaccines have prevented millions of individuals from acquiring otherwise life-threatening diseases. Although to date only smallpox and rinderpest have been wholly eradicated by global vaccination programs, vaccines have allowed countries to make significant strides toward potentially eradicating other diseases, such as polio and measles.^1^

While vaccination has played an immense role in improving public health, a growing number of individuals are actively choosing to forgo vaccination.^2^ These individuals have access to vaccination services but decide to either delay or refuse vaccination.^2^ In recent years, many studies have explored the emergence of this public health issue in patients; however, this growing problem has become increasingly common among health care providers.

A recent review by Majid and Ahmad^3^ discussed seven interconnected factors that influence childhood vaccine hesitancy amongst parents: past experiences; natural living; interactions with health care professionals; information sources; distrust in the health care system; and distrust in mandatory vaccination policies. A particularly intriguing finding from this study was that some parents believed that health care providers encouraged them to forgo childhood vaccination, which unintentionally promoted parental vaccine hesitancy. Another review of 185 studies found that health care providers who were either vaccinated themselves or were willing to vaccinate were more likely to recommend vaccination to patients.^4^ Paterson’s^4^ study identified the following factors that reduced health care providers’ vaccination recommendations to their patients: a belief that patients’ decision-making process should not be affected by their beliefs toward vaccines, low knowledge regarding vaccine efficacy and safety and being unprepared to discuss vaccines.

In contrast, a systematic review and meta-analysis by Vasilevska et al.^5^ found that vaccine acceptance amongst healthcare workers was linked to a desire for self-protection and protecting family and friends. This study also found that concern regarding vaccines transmitting illness was linked to decreased vaccine acceptance.^5^ However, to our knowledge, no study has specifically examined health care providers’ trust in the health care system, vaccine information, and vaccines. We believe that an investigation focusing on trust provides immense value to ongoing conversations and decisions about vaccination in health care providers. While trust cannot be separated from other issues that may contribute to vaccine hesitancy, we believe that the literature needs a more in-depth examination on how trust influences vaccine decision-making, which may further elaborate how trust works with other factors to encourage vaccine hesitancy. Our study delves into how health care providers’ trust in the health care system, employers, and other health care providers, may contribute to positive or negative views toward vaccines, and how trust in vaccines influences vaccine decision-making and provider recommendations to patients.

## Methods

### Search

In this review, we performed a systematic search utilizing a search strategy adapted from a previously published review on vaccine hesitancy in parents.^6^ This search strategy included three independent search filters that we used to narrow the search results: vaccines/immunization, attitudes and decision-making, and health care providers. We conducted the search on 26 September 2019, in the following databases: CINAHL, MEDLINE, Embase, PsychINFO, and the Web of Science (Supplementary file S1). We also conducted an updated search on 28 October 2020.

### Screening

We performed an initial screening of titles and abstracts, followed by a full-text screening of potentially relevant studies. We included English-language publications that met the eligibility criteria (table 1) and were qualitative or mixed-methods studies. Our screening was focused on any primary qualitative or mixed-method articles that mentioned any aspect of health care provider trust toward vaccines and vaccination. We included articles that mentioned findings or trust even if provider trust in vaccines was not the primary goal of the study.

**Table 1:** Eligibility criteria

Inclusion	Exclusion
English full-text publications Studies published after 2009 Primary, qualitative or mixed methods, empirical research (any descriptive or interpretive methodology) Studies that mention any aspect of trust concerning vaccines and vaccination, including mention of how trust may contribute to vaccine hesitancy Studies with the ‘primary focus’ on any component/aspect of vaccine hesitancy specific to a single vaccine (e.g. influenza) or specific to multiple vaccines, or does not specify a vaccine Peer-reviewed and published research work	Editorials, case reports, letters to the editor, or commentaries Work that has not been peer-reviewed or is not published or does not include primary qualitative data (e.g. theses) Work that is available in abstract or book chapter form only Studies that are labeled ‘qualitative’ but did not use a qualitative descriptive or interpretive methodology (e.g. experiments, surveys, or observational analyses using qualitative categorical variables) Ethical issues surrounding vaccine uptake, delivery, management, and delivery Articles that primarily focused on vaccine safety and efficacy but not specifically vaccine hesitancy in nurses and physicians

### Data extraction and analysis

We extracted several characteristics of the included articles utilizing a data extraction form. These characteristics included the title, research objectives, country, vaccination context, study setting, and participant demographics (i.e. field of practice, age, and ethnicity) (Supplementary file S1).

We conducted a systematic review and qualitative meta-synthesis analysis on included articles.^7^ This methodology allowed us to develop an integrative interpretation of physician and nurse trust in vaccines while ensuring that each study’s original meaning was retained. Initially, all three researchers analyzed five articles, and findings were recorded on analytic memos. We utilized these memos to develop a preliminary coding schema that served as a guide for coding the remaining articles. We modified the coding schema to incorporate novel findings every five articles. After analyzing the included articles, all researchers conducted focused coding, which involved reviewing coding from all articles to ensure that they were accurately organized based on themes and concepts. After focused coding, we employed the constant comparative method to explore the underlying meaning and context of themes present in the coding schema.^8^^,^^9^ We developed narrative summaries of all themes, which were then compiled and summarized by the lead author.

## Results

**Figure 1:**
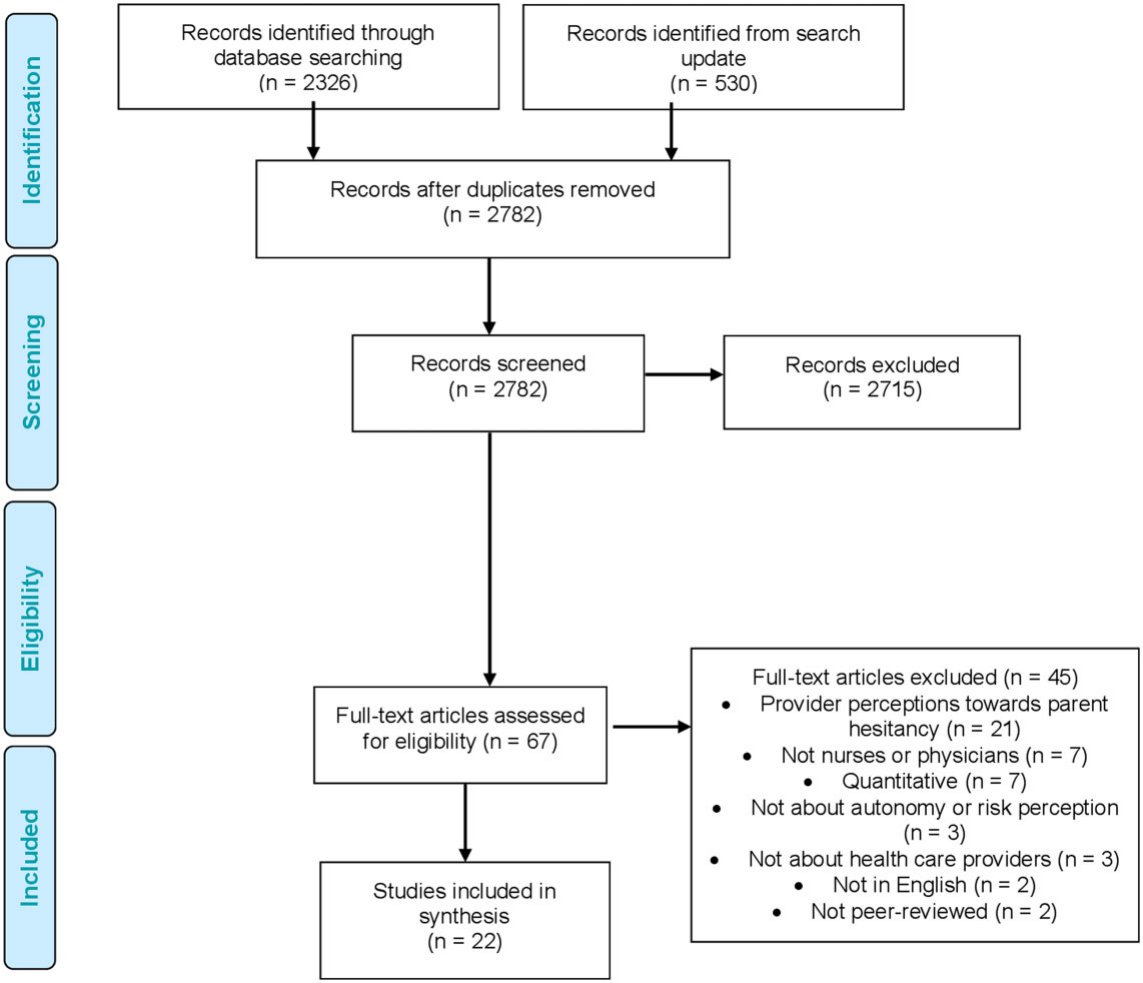
Screening and selection

We screened the title and abstracts of 2782 studies and excluded 2715. We then screened the full text of 67 studies and removed 47 for various reasons outlined in figure 1. In total, we analyzed a total of 22 studies in this review, of which 9 discussed vaccine hesitancy in physicians, 7 discussed vaccine hesitancy in nurses and 6 discussed vaccine hesitancy in both nurses and physicians. We have listed the countries of included studies in brackets wherever appropriate in the findings section. In many cases, studies were conducted in multiple countries, reflecting a diversity of physician and nurse experiences captured in our findings.

### Findings pertaining to nurses

Trust in vaccines played a significant role in nurses’ decision-making process. Vaccine-hesitant nurses expressed four factors that negatively influenced their trust in vaccines: distrust in health care authorities and their employers, distrust in new vaccines, distrust in vaccine content, and distrust because of vaccine side-effects (table 2).

**Table 2:** Summary of findings (nurses)

Theme	Findings
Distrust in health authorities, employers and pharmaceutical companies	Nurses felt that physicians were influenced by pharmaceutical companies to promote vaccination through clandestine relationships and financial incentives.^10^^,^^12^^,^^14^^,^^16^ Aggressive tactics utilized by employers made nurses feel their health was a lower priority to employers than the health of patients.^12^^,^^14^^,^^17^ Nurses were skeptical of information provided by employers, health authorities and pharmaceutical companies due to negative interactions they had with health authorities and since the information did not address their specific concerns.^10–^^13^^,^^17–19^
Distrust in vaccine efficacy and contents	Nurses were mistrustful of newly developed vaccines as they believed that they underwent fewer tests and had more side-effects than previously developed vaccines.^10^^,^^13^^,^^16^^,^^19^^,^^20^ Some nurses believed vaccines were not efficacious, causing them to question whether vaccinating was in their best interests.^12^^,^^17^^,^^19^^,^^20^ Nurses expressed concerns regarding various vaccine side-effects including allergies, sinusitis, fever, joint pain, fatigue and flu.^11^^,^^12^^,^^19^^,^^22^ Nurses in included studies had concerns regarding the safety of vaccine ingredients and manufacturing processes.^12^^,^^13^^,^^19^^,^^22^

#### Distrust in health authorities, employers and pharmaceutical companies

In four of the included studies (Cyprus, Germany, Greece, Israel, Italy, Lithuania, Poland, Romania, Switzerland and USA), nurses expressed distrust in the motives of their employers (i.e. physicians, hospitals and clinics);^10–13^ in two studies (Cyprus, Germany, Greece, Italy, Lithuania, Poland, Romania and USA) they were distrustful of health authorities (i.e. medical boards and the government);^11^^,^^16^ and in three studies (Germany and USA) they were distrustful of pharmaceutical companies^12^^,^^15^^,^^16^ for promoting vaccination in the workplace. Common perceptions held by nurses that reinforced distrust in their employers and health authorities included the following: pharmaceutical companies provided financial incentives to physicians to promote vaccination; the health of nurses was not explicitly emphasized and thus unimportant; and health authorities provided inaccurate and inconsistent vaccine information.

Nurses felt that physicians were influenced by pharmaceutical companies to promote vaccination through clandestine relationships and financial incentives: ‘from my point of view all it’s really about the money. It's not about the patient… I think there's a Mafia between the doctors and the pharma industry’.^12^ Nurses felt that pharmaceutical companies were primarily interested in financial gain rather than patient health, and this perception engendered mistrust amongst nurses toward vaccines.^12^^,^^14^^,^^15^ Additionally, some nurses felt that vaccines were intentionally promoted to them by pharmaceutical companies and physicians to further test the safety of vaccines before introducing it to the general public.^10^ Due to these ulterior motives, nurses felt that it was challenging to trust vaccine recommendations from physicians. Alongside ulterior motives, certain coercive tactics used by employers to pressure nurses to vaccinate made them feel that their health was a lower priority than the health of patients; nurses felt aggressive tactics were used to vaccinate them instead of involving them in conversations about the need and importance of vaccination.^12^^,^^14^^,^^17^ These tactics bolstered nurses’ beliefs that patient health was prioritized over their health as they focused on the benefits vaccines had on patients rather than nurses: ‘the other thing that has always bothered me is those campaigns that have been made…that you basically have to have a bad conscience if you don't get vaccinated…’.^12^ Nurses expressed how vaccination campaigns put an intense pressure on them to vaccinate that led to feelings of guilt if they chose not to vaccinate.^12^ Side-effects that nurses experienced after receiving vaccinations from physicians strengthened their view that they were a lower priority than other health care providers and patients; nurses felt that their concerns were not recognized and often ignored when they experienced side-effects.^10^^,^^12^^,^^14^

Another factor contributing to nurses’ distrust in health authorities and employers was the nature of information on vaccines by reputable allopathic medicine sources.^10^^,^^11^^,^^13^ Firstly, nurses cited prior negative interactions with health authorities as a reason for their distrust in them.^10^^,^^12^^,^^17^^,^^18^ Nurses mentioned how their interactions with health authorities during times of pandemics increased their distrust toward health authorities, primarily because of the inconsistent information that nurses received: ‘with the swine flu there was a lot of disinformation at the beginning, there was a lot of confusion at the beginning’.^10^ This quote shows how some nurses felt the information was unclear or even false. Nurses in included studies also felt that frequent changes to vaccine information and guidelines added to their prior perception of information provided by health authorities as inconsistent.^10^^,^^15^ Nurses felt that inconsistent vaccine information was a health risk to them because vaccination may later be proven detrimental to their health, which increased their distrust in information sources.^10^

Vaccine-hesitant nurses in three studies also believed that allopathic vaccine information did not address their concerns regarding vaccines and vaccination.^10^^,^^17^^,^^19^ Nurses emphasized the need for information regarding the efficacy, side-effects and complications of certain vaccines to help them make an informed vaccination decision.^19^ As a result, some nurses were unaware of the safety profile and efficacy of vaccines, causing an overestimation of the potential risks with vaccination.^19^

#### Distrust in new vaccines

In addition to distrust in health authorities and employers, nurses in eight studies (Australia, Canada, Cyprus, Germany, Greece, Israel, Italy, Poland, Romania and Lithuania) were mistrustful of vaccines and their health benefits.^10–14^^,^^20^^,^^21^ Commonly expressed concerns regarding vaccines by nurses included mistrust of newer vaccinations, mistrust in vaccine efficacy, concerns regarding vaccine side-effects and concerns regarding vaccine content.

Nurses in five studies (Canada, Israel and USA) expressed greater mistrust in newer vaccines than those that had been available to the public for years.^10^^,^^13^^,^^16^^,^^19^^,^^20^ The vaccines often referred to as new in these studies included the HPV vaccine, the H1N1 vaccine and the annual influenza vaccine.^10^^,^^13^^,^^16^^,^^19^^,^^20^ This mistrust stemmed primarily from a perception that newer vaccines had more side-effects due to the lack of testing: ‘you know in the past there was, not problems, but probably more side-effects with some of the vaccines, just because they were newer, and anyway they refined them…’.^20^ Nurses believed that vaccines have been refined to counteract their side-effects overtime; a characteristic that did not exist for new vaccines.^20^ At the same time, nurses believed that research regarding the long-term side-effects of newer vaccines was unavailable to them.^13^^,^^19^^,^^20^

#### Distrust in vaccine content

A concern related to distrust in new vaccine was regarding the makeup and ingredients in vaccines mentioned by nurses in four studies (Greece, Switzerland and USA).^12^^,^^13^^,^^19^^,^^22^ Since vaccines were produced rapidly to meet public demand, nurses believed that manufacturers were unable to properly ensure the safety of vaccines: ‘I also don't believe that it's always pure and stuff because it's always done fast, under pressure. Everyone has to be the first, that's the one who can put it on the market. And like with everything that happens in a hurry…there's no more regard for thoroughness, for cleanliness’.^12^ Nurses may extrapolate their concerns regarding vaccine manufacturing process to all vaccines thereby increasing their hesitancy to vaccinate.^12^

#### Distrust because of vaccine side-effects

Nurses in six included studies expressed concerns regarding a multitude of vaccine side-effects.^11^^,^^12^^,^^15^^,^^16^^,^^19^^,^^22^ Nurses mentioned a number of side-effects that they believed to be associated with vaccines, including allergies, sinusitis, fever, joint pain, fatigue and flu.^11^^,^^12^^,^^19^^,^^22^ Nurses concerns stemmed from personal experiences with side-effects they believed to be associated with vaccines: ‘I took one [flu shot] a couple of years ago and my whole family got the flu. I didn't take one last year, and we never got it’.^13^ This quote demonstrates the repercussions of perceived vaccine side-effects in a precarious situation where the nurse’s family acquired flu only after vaccination but avoided the flu when they did not vaccinate, which led to complete avoidance of vaccination in the future.^16^ Mistrust in the safety of vaccines may have been reinforced by the fact that the nurse’s family remained healthy after the decision to not vaccinate. Exacerbating this situation, nurses may extrapolate their understanding of the side-effects of one vaccine to strengthen their decision to avoid other vaccines in the future.

### Findings pertaining to physicians

#### Mistrust toward pharmaceutical companies

Physicians in the included studies (Canada, Croatia, Cyprus, France, Germany, Greece, Italy, Lithuania, Poland, Romania and USA) expressed their mistrust of the vaccine industry due to the financial motivations of pharmaceutical companies.^11^^,^^14^^,^^15^^,^^20^^,^^23^^,^^24^ In one study, physicians mentioned how they felt pressured by the pharmaceutical industry to promote certain vaccines without fully knowing their potential side-effects.^24^ Some even recalled how company representatives would persuade physicians to use one vaccine over the other to increase their profit margins.^24^ The privileging of certain vaccines, coupled with miscommunication regarding their potential side-effects, was enough to promote mistrust surrounding the underlying motives of pharmaceutical companies (table 3).^24^

**Table 3:** Summary of findings (physicians)

Theme	Findings
Mistrust toward pharmaceutical companies	Physicians expressed mistrust toward the vaccine industry due to the financial motivations of pharmaceutical companies.^11^^,^^14^^,^^15^^,^^20^^,^^23^^,^^24^ Physicians felt pressured by pharmaceutical companies to promote certain vaccines without knowing the full extent of potential side-effects.^24^
Vaccine safety, efficacy and side-effects	Physicians were highly concerned about the overall quality and safety of vaccines.^11^^,^^20^^,^^21^^,^^24–29^ Personal as well as patient experiences with vaccine side-effects have played a critical role in bolstering sentiments of hesitancy.^11^^,^^15^^,^^29^^,^^30^ Some physicians were adamant about possible serious vaccine side-effects with some believing that they caused debilitating diseases, such as Multiple Sclerosis and cancerous tumors.^24^

#### Vaccine safety, efficacy and side-effects

Physicians were highly concerned about the overall quality and safety of several vaccines across nine studies (Australia, Canada, Croatia, Cyprus, England, France, Germany, Greece, Israel, Italy, Lithuania, the Netherlands, Poland, Romania and USA).^11^^,^^20^^,^^21^^,^^24–29^ In five studies, physicians doubted the efficacy of the influenza vaccine.^11^^,^^15^^,^^20^^,^^26^^,^^29^ For example, even when presented with scientific evidence supporting the administration of the influenza vaccine, primary care physicians in one study still doubted its effectiveness.^26^ Despite these beliefs, primary care physicians continued to administer the vaccine as they felt it was inappropriate to violate government mandated vaccine programs.^26^ Other primary care physicians also generally agreed with the administration of vaccines, trusting that the government had done sufficient testing and research to justify its safety and efficacy in the population.^26^ This is stark in contrast to another included study where physicians did not trust the Greek government’s vaccine policies.^11^ Mandatory vaccination policies were particularly mistrusted because some physicians felt they infringed on their autonomy.^11^ Because of intense distrust in the government, physicians dissuaded their patients from vaccination.^11^ Personal as well as patient experiences with vaccine side-effects have played a critical role in bolstering sentiments of hesitancy.^11^^,^^15^^,^^29^^,^^30^ A fear of inciting vaccine side-effects amongst patients was so much so that physicians in one study stated liability as a primary reason for not promoting vaccines.^31^

Many physicians in one study were adamant about possible serious vaccine side-effects with some believing that they caused debilitating diseases, such as Multiple Sclerosis and cancerous tumors.^24^ This belief often originated from physicians’ interactions with patients who believed that vaccines led to the emergence of these diseases in themselves or close acquaintances.^25^ Communication between physicians and patients played an important role in promoting vaccine hesitancy in patients.^24^^,^^25^ Some physicians felt guilty if their patients suffered side-effects from a vaccine they administered.^24^

## Discussion

### Review of findings

Our study explored how trust plays a crucial role in shaping physician and nurse perceptions toward vaccines. In the following sections, we use the concept of unsystematic clinical experiences (i.e. poorly documented, and uninvestigated experiences with managing patient care) to identify the reasons that may contribute to vaccine hesitancy in nurses and physicians. We also highlight similarities and differences between our findings on nurses and physicians, including how some aspects of the nurse–physician relationship parallel the parent–physician relationship with regards to conversations on vaccination.

### Evidence-based medicine and unsystematic clinical experiences

Evidence-Based Medicine (EBM) is defined as the ‘conscientious, explicit, judicious and reasonable use of modern, best evidence in making decisions about the care of individual patients’.^32^ EBM utilizes a combination of clinical experience and patient values with the best available research to provide the best care for patients.^33^ In recent years, EBM has been upheld as the ‘gold standard’ of clinical decision-making.^34^ All three components play an important role in clinical decision-making. However, before EBM, health care providers predominantly used ‘unsystematic clinical experiences’ (i.e. poorly documented, and uninvestigated experiences with managing patient care) to expand their knowledge of patient prognosis, treatment efficacy and diagnostic test value.^33^

Unfortunately, health care providers may still use unsystematic clinical experiences in clinical decision-making,^35^ and this may cause certain issues for health care providers who are reluctant to vaccinate themselves. While health care providers’ utilization of unsystematic clinical experience in patient management has been studied previously, there has been insufficient research to our knowledge on the impact of unsystematic clinical experience on health care providers’ trust in vaccination. Our study’s findings indicate that health care providers often use previous unsystematic workplace and clinical experiences—specifically the negative experiences that they or their patients have had with vaccines—to communicate their trust and vaccine recommendations to patients. In the following sections, we discuss how nurses’ and physicians’ negative interactions may contribute to unsystematic clinical experiences that shape their vaccination perceptions and promote vaccine hesitancy.

### Nurse and physician clinical experiences

Nurses in included studies demonstrated a decreased trust in vaccination after experiencing side-effects that they attributed to vaccines. Experiencing the possible side-effects of one vaccine may also engender suspicion regarding the safety of other vaccines. Attributing side-effects to vaccines acted as an unsystematic clinical experience for nurses that further increased vaccine hesitancy.^10^^,^^12^^,^^14^

Intensifying this unsystematic clinical experience was nurses’ negative interactions with physicians after experiencing potential vaccine side-effects, where they felt that their symptoms and concerns were not being recognized.^10^^,^^12^^,^^13^ These interactions between nurses and physicians paralleled interactions between vaccine-hesitant parents and physicians.^36^ For example, in-depth interviews conducted by Carrion et al.^36^ of 50 vaccine-hesitant mothers in the USA found that like nurses, parents explained how physicians ignored their vaccine side-effect concerns, bolstering their vaccine hesitancy. Without acknowledging and addressing nurses’ vaccination concerns, nurses were left to assess the safety and side-effects of vaccines by themselves, which may have led to the strengthening of pre-existing vaccine hesitancy.

The impact of addressing individuals’ vaccination concerns can be seen in Shay et al.’s^37^ study during which they interviewed 43 parents hesitant about the HPV vaccine after they discussed the vaccine with their physician. They found that having an exclusively persistent approach to the parent–physician interaction in which physicians emphasized the importance of the vaccine, and probed to understand and address parental questions and concerns regarding the vaccine led to 17 out of 18 adolescents being vaccinated that office visit.^37^

Similarly, physician trust in vaccines was also influenced by the unsystematic clinical experiences pertaining to the potential vaccine side-effects in their patients. We found that unsystematic clinical experiences have a strong influence on physicians’ beliefs or views toward vaccination because in some cases they opposed years of medical education and practicing EBM.^11^^,^^12^^,^^29^^,^^30^

Like physicians and nurses, unsystematic clinical experiences also mold perceptions of vaccines amongst the general population. An online Polish cross-sectional survey of 492 vaccine-hesitant individuals found that 47% stated that they believed they had negative side-effect to vaccines.^38^ These individuals were notably more supportive of anti-vaccination activists, more likely to believe that vaccines had detrimental side-effects and held more doubts about vaccine efficacy and the intentions of healthcare providers as compared to other study participants.^38^ Several reasons may explain physician tendency to prioritize unsystematic clinical experiences, including lack of up-to-date information about the pros and cons of vaccination, exposure from strong social circles that promote vaccine hesitancy and the recency bias.

### Implications of this research

In addition to describing why many health care providers are vaccine hesitant, our findings also provide insight into the possible interventions that may address provider vaccine concerns. With global COVID-19 vaccination programs underway, it is essential that we address health care providers’ concerns to ensure that they will promote vaccination to patients. A recent web-based survey of Turkish physicians conducted by Civelek et al.^39^ found that 24% of physicians were undecided regarding vaccination for COVID-19 and 7.6% stated they would not receive the vaccine. Their study highlighted the importance of addressing the growing issue of provider vaccine hesitancy.

Nurses in our study were especially expressive of the lack of recognition by physicians and employers of possible vaccine side-effects they may have experienced.^10^^,^^12^^,^^14^ Countries around the world have systems in place to report vaccine side-effects. Examples of these systems include the Vaccine Adverse Event Reporting System (VAERS) in the USA, and the Canadian Adverse Events Following Immunization database.^40^^,41^ Reporting to these systems, however, is often voluntary, which may therefore contribute to underreporting. The VAERS website acknowledges underreporting as a challenge and argues that datasets may be subject to biases of those that report the information.^40^ In order to have accurate information regarding vaccine safety, continuing medical education that informs providers on how to discern between a vaccine reaction and another medical condition is necessary. This may include educating healthcare providers on how some patients may attribute symptoms to vaccines, which may be unrelated to their recent vaccination (i.e. the nocebo effect).^42^ Additionally, there must be incentives that encourage health care provider to report these events. This may be accomplished by adding a prompt in electronic medical record systems that asks health care providers if a patient suffered from a vaccine reaction during follow-up appointments.

Interventions aimed at creating a non-judgmental environment allowing for two-way communication between nurses, physicians, employers and health authorities needs to be established to address nurses’ concerns. Such an environment may be possible through the development of a vaccine information hotline, such as the one utilized by the Australian government.^43^ These hotlines allow for individuals to ask questions regarding their vaccination concerns anonymously and in the comfort of their homes. Having these information outlets available to them may decrease the likelihood of nurses experiencing negative interactions with other health care providers, thereby reducing the impact of unsystematic clinical experiences on patients. These interventions can also create a space for healthcare providers and patients to understand that not all symptoms experienced are associated with vaccination.

## Supplementary data

Supplementary data are available at *EURPUB* online.

## Disclaimer

Neither party was involved in the design and conduct of this research.

**Conflicts of interest:** U.M. receives financial support from the Canadian Institutes of Health Research and the Government of Ontario, Canada. Neither party was involved in the design or conduct of this research.

Key points

- This study explores the factors that increase vaccine distrust amongst physicians and nurses.
- Factors that negatively influenced nurses’ trust in vaccines included distrust in health care authorities and their employers, and distrust in vaccine efficacy.
- Physicians held similar concerns regarding pharmaceutical companies and their motives, as well as vaccine efficacy and side-effect.
- Addressing these concerns is essential for ensuring that physicians and nurses promote COVID-19 vaccines to their patients.

## Supplementary Material

ckab209_Supplementary_Data
