# Supplementary material for: Health care provider trust in vaccination: a systematic review and qualitative meta-synthesis
Source: Eur J Public Health. 2022 Jan 11;32(2):207–13. doi: 10.1093/eurpub/ckab209 (PMC9090277; doi:10.1093/eurpub/ckab209)
Supplement: ckab209_Supplementary_Data [file ckab209_supplementary_data.docx]

**Health Care Provider Trust in Vaccination: A Systematic Review and Qualitative Meta-Synthesis**

Supplementary File

[Search Strategy 2](#_Toc82697574)

[Additional References 3](#_Toc82697575)

[Study and Methodological Characteristics of Included Studies 4](#_Toc82697576)

[Demographic Characteristics of Participants 9](#_Toc82697577)

# Search Strategy

**MEDLINE** (via Ovid)

September 24, 2019

1/ exp *immunization/ or exp *vaccines/ or exp *"immunization programs"/

2/ (immuni#ation* or immuni#e* or immuni#ing or vaccin* or anti-vaccin* or antivaccin* or pro-vaccin* or provaccin*).ti,ab.

3/ 1 or 2

4/ attitude/ or exp "attitude to health"/ or "choice behavior"/ or "consumer behavior"/ or culture/ or "decision making"/ or exp "health behavior" or knowledge/ or motivation/ or "public opinion"/ or "refusal to participate"/ or "rejection (psychology)"/ or "social determinants of health"/ or "social norms"/ or "social perception"/ or "social values"/ or trust/ or uncertainty/

5/ (accept* or adherence or attitude* or barrier* or behavio?r* or belief* or choice* or compliance or confidence or cultur* or decision* or determinant* or dislik* or distrust* or doubt* or dropout* or drop-out* or exemption* or expectation* or experience* or fear* or hesita* or intention* or knowledge or mistrust* or motivat* or nonadherence or non-adherence or noncompliance or non-compliance or opinion* or participat* or perception* or practice* or preference* or reason* or reluctan* or refus* or satisfaction or trust* or uptak* or view* or willing*).ti,ab.

6/ 4 or 5

7/ 3 and 6

8/ exp Nurse Clinicians/ or exp Nurse Practitioners/ or exp Nurse Administrators/ or nurse*.mp. or exp Nurse Midwives/ or exp Nurse Specialists/

9/ exp Physician Executives/ or exp Physician Assistants/ or physician*.mp.

10/ exp Health Personnel/

11/ health care worker$.mp.

12/ healthcare worker$.mp.

13/ exp Complementary Therapies/

14/ alternative therap*.mp.

15/ complementary medicine.mp.

16/ medicine alternative$.mp.

17/ alternative medicine$.mp.

18/ or/8-17

19/ "focus groups"/ or "interviews as topic"/ or exp narration/ or observation/ or "qualitative research"/ or "grounded theory"/

20/ (("semi-structured" or semistructured or unstructured or informal or "in-depth" or indepth or "face-to-face" or structured or guide) adj2 (interview* or discussion* or questionnaire*) or ethnograph* or fieldwork* or (focus adj group*) or (group adj discussion*) or observation* or (grounded adj theor*) or ((document* or thematic) adj analysis)).ti,ab.

21/ 19 or 20

22/ 7 and 18 and 21

23/ 22 not (exp animals/ not humans/)

24/ 23 not (news or comment or editorial).pt

25/ 24 and (English or French).lg [988]

# Additional References

41. Canadian Adverse Events Following Immunization Surveillance System (CAEFISS) [Internet]. Government of Canada;[cited 2021 Feb 11]. Available from: <https://www.canada.ca/en/public-health/services/immunization/canadian-adverse-events-following-immunization-surveillance-system-caefiss.html>

42. Planès S, Villier C, Mallaret M. The nocebo effect of drugs. Pharmacology research & perspectives. 2016 Apr;4(2):e00208.

43. National Immunisation Information Line [Internet]. Australian Government Department of Health;[cited 2021 Feb 11]. Available from: https://www.health.gov.au/contacts/national-immunisation-information-line

# Study and Methodological Characteristics of Included Studies

| **Author (Year)** | **Title** | **Objectives** | **Country** | **Study Setting** | **Design** | **Data Collection Method** |
| --- | --- | --- | --- | --- | --- | --- |
| Barron-Epel (2012) | What lies behind the low rates of vaccinations among nurses who treat infants? | Identify reasons behind the low rates of compliance of Israeli nurses in Mother and Child Health care Centers (MCHC) with an official request for pertussis vaccination. | Israel | Urban | Thematic Analysis | Focus Groups |
| Bean (2013) | Vaccine Perceptions Among Oregon Health Care Providers | Examine antecedents of pediatric vaccine recommendations among different types of health care providers (HCPs) in the state of Oregon | United States of America | NR | Thematic Analysis | Semi-structured Interviews |
| Biezen (2018) | Why do we not want to recommend influenza vaccination to young children? A qualitative study of Australian parents and primary care providers | Explore the views, attitudes and practices of parents and primary care providers (PCPs) on their knowledge and acceptance of influenza vaccination in children under 5 | Australia | Urban | Thematic Analysis | Interviews and Focus Groups |
| Dalma (2018) | Promotion of Immunizations for Health Professionals in Europe: A Qualitative Study in Seven European Member States | Understand their barriers to vaccination and needs of HCWs with regards to vaccination. | Italy, Greece, Cyprus, Romania, Germany, Poland and Lithuania | Urban | Constant Comparative Method and Grounded Theory | Focus Groups |
| Edge (2017) | Socialization, Indifference, and Convenience: Exploring the Uptake of Influenza Vaccine Among Medical Students and Early Career Doctor | Explore the factors informing their (HCPs) influenza vaccination decision making | England | NR | Thematic Analysis | Semi-structured Interviews |
| Eilers (2015) | Attitudes of Dutch general practitioners towards vaccinating the elderly: less is more? | Explore Dutch GPs’ attitudes regarding vaccination in general, and their attitudes regarding the incorporation of additional vaccines in the current Dutch influenza vaccination programme | Netherlands | NR | Thematic Analysis | Interviews |
| Flanagan (2020) | Barriers and facilitators to seasonal influenza vaccination  uptake among nurses: A mixed methods study | Identify the barriers and facilitators to seasonal influenza vaccination uptake  among nurses | Ireland | NR | Thematic Analysis | Focus Groups |
| Gesser-Edelsburg (2017) | Despite awareness of recommendations, why do health care workers not immunize pregnant women? | Assess the views of healthcare workers regarding conveying recommendations for pregnant women to immunize against Tdap and influenza. | Israel | Urban | Inductive Content Analysis | Questionnaires |
| Gonthier (2020) | Postponing vaccination in children with an  infection: a qualitative study among general  practitioners and pediatricians | Explore the factors motivating the practice of vaccination  postponement in a child with an infection by general practitioners (GPs) and paediatricians | France | Both | Grounded Theory | Semi- directive Individual Interviews |
| Hagemeister (2018) | Self-reported influenza vaccination rates and attitudes towards vaccination among health care workers: results of a survey in a German university hospital | Analyse vaccination rates and attitudes towards vaccination among health care workers (HCWs) | Germany | Urban | Grounded Theory | Questionnaires |
| Kahn (2007) | Factors Influencing Pediatricians’ Intention to Recommend Human Papillomavirus Vaccines | Describe the range of pediatricians’ attitudes about human papillomavirus (HPV) vaccines and to explore factors influencing their intention to recommend HPV vaccines, extending the findings of previous quantitative studies | United States of America | Urban | Framework Analysis | Semi-structured interviews |
| Karafillakis (2016) | Vaccine hesitancy among healthcare workers in Europe: A qualitative study | Understand vaccine hesitancy among vaccine providers in Europe, and explore the nature of their concerns, their perceptions of vaccine-related information, and their perceived role in responding to vaccine hesitancy | Croatia, France, Greece, and Romania | NR | Thematic Analysis | Semi-structured Interviews |
| Leaske (2010) | Making influenza vaccination mandatory for health care workers: the views of NSW Health administrators and clinical leaders | Understand the views of NSW Health administrators and clinical leaders about adding influenza vaccination to the requirements | Australia | NR | Thematic Analysis | Semi - structured Interviews |
| Manca (2016) | Health professionals and the vaccine narrative:  ‘the power of the personal story’ and the  management of medical uncertainty | 1) Explore the sociological understandings of medical uncertainty and medical progress 2) Explore how the creation of an 'illusion of uncertainty' in the health profession relates to the vaccine narrative 3) Review how various disease outbreaks in Alberta have been attributed to patients’ vaccine  uncertainties 4) Analyse some of the uncertainties that professionals experienced and the  tactics they used to manage uncertainties and knowledge gaps about vaccines | Canada | Both (Urban & Rural) | Narrative & Image Analysis | Interviews |
| Manuel (2002) | Health Behavior Associated with Influenza Vaccination Among Health Care Workers in Long-Term–Care Facilities | Investigate the health behavior associated with influenza vaccination among healthcare workers (HCWs)in long-term–care facilities. | Canada | NR | Thematic Analysis | Cross-Sectional Surveys and Focus Groups |
| Perkins (2012) | What Affects Human Papillomavirus Vaccination Rates? A Qualitative Analysis of Providers’ Perceptions | Define factors that providers perceive as affecting their administration of human papillomavirus (HPV) vaccination in their clinical practices. | United States of America | Urban | Grounded-theory | Semi-structured interviews |
| Pless (2017) | Reasons why nurses decline influenza vaccination: a qualitative study | Explore reasons of non-vaccinated nursing staff for declining seasonal influenza vaccination | Switzerland | NR | Content Analysis | Semi-structured Interviews |
| Raftopoulos (2008) | Attitudes of nurses in Greece towards influenza vaccination | Explore the attitudes and beliefs of nurses towards influenza vaccination in Greece | Greece | NR | Content Analysis | Focus Groups |
| Rhudy (2010) | Personal Choice or Evidence-Based Nursing Intervention: Nurses’ Decision-Making about Influenza Vaccination | Understand the factors influencing nurses’ decision-making about personally receiving immunization against influenza | United States of America | Urban | Content Analysis | Semi - structured interviews |
| Tuckerman (2016) | Understanding motivators and barriers of hospital-based obstetric and pediatric health care worker influenza vaccination programs in Australia | Explore key drivers and HCW decision making related to recommended vaccines and seasonal influenza vaccination programs | Australia | Urban | Thematic Analysis | Semi-structured Interviews |
| Willis (2007) | Nurses’ attitudes and beliefs about influenza and the influenza vaccine: A summary of focus groups in Alabama and Michigan | Explore the attitudes and beliefs of nurses towards influenza vaccination because information specific to nurses is limited | United States of America | Urban | Thematic Analysis | Focus Groups |
| Wilson (2020) | Vaccine hesitancy among general practitioners in Southern France and their  reluctant trust in the health authorities | Understand vaccine hesitant GPs’ views towards vaccines | France | Both | Thematic Analysis | Telephone Questionnaire and Semi-structured Interviews |

# Demographic Characteristics of Participants

| **Author (Year)** | **Vaccination Context (full support, partial support or rejection)** | **Vaccine of Interest** | **Total Number and Profession of Healthcare Providers** | **Medical Disciplines of Healthcare Providers** | **Proportion of Males and Females** | **Age Range** |
| --- | --- | --- | --- | --- | --- | --- |
| Barron-Epel (2012) | NR | Pertussis | 25 Nurses | NR | NR | NR |
| Bean (2013) | Full support, partial support and rejection | NR | 15 HCPs (4 allopathic or osteopathic physicians/pediatricians, 2 nurses, 9 CAMs including chiropractors and midwives) | Pediatrics, General Practice, Midwifery and Chiropractics | 9 Females & 6 Males | 34-70 |
| Biezen (2018) | NR | Influenza | 30 Primary Care Providers (20 GPs, 2PNs, 3 MCHNs and 5 Pharmacists) | General Practice, Maternal & Child Health and Pharmaceutics | NR | 25-60 |
| Dalma (2018) | Partial support and rejection | NR | 278 HCPs (physicians, nurses, hospital infection control personnel, and public health personnel); amount of each not specified | NR | NR | NR |
| Edge (2017) | Full support, partial support and rejection | Booster (MMR, varicella, hepatitis B, Td, Tdap vaccines), seasonal and pandemic influenza | 7 Medical students and 9 residents | NR | NR | NR |
| Eilers (2015) | Full support, partial support and rejection | NR | 10 General Practitioners | General Practice | 5 Females & 5 Males | NR |
| Flanagan (2020) | Full support and rejection | Influenza | 462 Nurses | NR | 426 Females & 33 Males; 3 NR | 20-29; >50 |
| Gesser-Edelsburg (2017) | Full and partial support | Tdap and Influenza | 119 HCPs (81 Gynecologists and 38 Family Practitioners) | Gynecology and Family Medicine | 84 Females & 64 Males; 2 NR | 22-67 |
| Gonthier (2020) | NR | NR | 18 Physicians (14 GPs and 4 Pediatricians) | General Practice and Pediatrics | 10 Females & 8 Males | NR; mean age of 46 |
| Hagemeister (2018) | Full support, partial support and rejection | Influenza | 664 HCPs (422 Nurses and 242 Doctors) | NR | 488 Females & 180 Males | 16-65 |
| Kahn (2007) | Full support | HPV | 31 Pediatricians | Pediatrics | 17 Females & 14 Males | 30-78 |
| Karafillakis (2016) | Partial support and rejection | NR | 65 HCPs (GPs – 72%, GYN. – 9%, Epidemiologists – 6%, Pediatrician – 6%, Internal Medicine Specialists – 6%) | General Practice, Gynecology, Epidemiology, Pediatrics & Internal Medicine | 43 Females & 22 Males | 25-44; 46-65; >65 |
| Leaske (2010) | Full support, partial support and rejection | Influenza | 37 clinicians, clinical managers and administrative leaders; 8 NR | NR | NR | NR |
| Manca (2016) | Full support | NR | 26 Physicians & 7 Nurses | NR | NR | 30-70 |
| Manuel (2002) | Full support, partial support and rejection | Influenza | 44 Nurses, 85 Healthcare Aides | NR | 206 Females & 23 Males | <30; 30-39; 44-49; >50 |
| Perkins (2012) | Full and partial supporter and rejection | HPV Vaccine | 18 Pediatricians, 8 Family physicians, & 8 nurse practitioners | Pediatrics & Family Medicine | 28 Females & 6 Males | NR |
| Pless (2017) | Rejection | Influenza | 18 Nurses | Hematology, Cardiology, Nephrology, Geriatrics, ICU & Oncology | 14 Females & 4 Males | NR |
| Raftopoulos (2008) | Partial support and rejection | Influenza | 30 nurses | NR | 23 Females & 7 Males | NR |
| Rhudy (2010) | Partial supporters and rejection | Influenza | 14 Registered Nurses | NR | 11 Females & 2 Males | 25-61 |
| Tuckerman (2016) | Full support and rejection | Influenza | 22 HCWs (14 RNs,5 Midwives,2 MDs, one administrative officer) | Midwifery, others NR | 21 Females & 1 Male | NR |
| Willis (2007) | Partial support and rejection | Influenza | 71 Registered Nurses | NR | NR | NR |
| Wilson (2020) | Rejection | NR | 19 General Practitioners | General Practice | 11 Females & 8 Males | 36-45; 46-66; 56-65; >65 |
